# Initial Molecular Detection of Membrane Damage in Post-Golgi Compartments

**DOI:** 10.3390/cells15151375

**Published:** 2026-07-30

**Authors:** Yoko Shiba, Nana Saito

**Affiliations:** Graduate School of Science and Engineering, Iwate University, 4-3-5, Ueda, Morioka 020-8551, Japan

**Keywords:** membrane damage, galectin, ESCRT, sphingomyelin, PI4P, annexins, membrane repair by fusion

## Abstract

**Highlights:**

**What are the main findings?**

- Initial membrane damage is detected through multiple mechanisms, including galectins, ESCRT, sphingomyelin exposure, PI4P-associated pathways and annexins.
- Studies of the plasma membrane, lysosomes, and bacteria-containing vacuoles provide insights into endosomal membrane damage.

**What are the implications of the main findings?**

- Ca^2+^ influx provides a framework for damage sensing; however, multiple Ca^2+^-independent mechanisms also contribute to damage sensing and membrane repair.
- Understanding membrane damage will facilitate the development of drug delivery systems and physical-energy based intracellular delivery technologies.

**Abstract:**

Mammalian cells contain numerous membrane-bound organelles, of which endosomes serve as the initial destination for endocytosed molecules. Therapeutic agents are also internalized by cells and transported to endosomes or phagosomes and subsequently delivered to lysosomes for degradation. Therefore, these agents require drug delivery systems (DDSs) that enable their escape from endosomes into the cytosol before lysosomal degradation; however, endosomal escape is a major limitation of current DDSs. Studies of bacterial phagosomal escape have revealed mechanisms by which host cells detect damage to organelle membranes. These membrane damage-sensing molecules also recognize membrane damage caused by artificial DDSs or physical energy-based insults. In this review, we summarize the molecular mechanisms underlying the early stages of membrane damage in the plasma membrane, lysosomes and bacteria-containing vacuoles (BCVs) to better understand the early stages of endosomal membrane damage in the absence of pathogens. We summarize recent advances in galectins, endosomal sorting complexes required for transport (ESCRT) complexes, sphingomyelin, stress granules, and phosphatidylinositol 4-phosphate (PI4P) at membrane contact sites, as well as annexins. We also discuss the recruitment kinetics of these molecules to damaged membranes. Although the recruitment kinetics vary depending on cell type and experimental conditions, this information provides a timeframe for the events following membrane damage, including damage sensing, membrane repair, and degradation of damaged organelles. We also discuss a potential fourth event, fusion between the plasma membrane and endosomes or lysosomes for membrane repair in the annexin section. Finally, we summarize approaches for inducing “sterile” endosomal membrane damage. Future development of these approaches may facilitate the design of novel DDSs and physical energy-based strategies for manipulating specific organelles.

## 1. Introduction

Mammalian cells internalize nutrients, solutes, growth factors, and hormones that bind to specific receptors on the plasma membrane through endocytosis. These cargoes are internalized along with their receptors. Several endocytic pathways have been described, including clathrin-mediated endocytosis (CME), fast endophilin-mediated endocytosis (FEME) (clathrin-independent but dynamin-dependent), clathrin-independent carrier (CLIC)/glycosylphosphatidylinositol-anchored protein-enriched early endocytic compartment (GEEC) endocytosis, macropinocytosis, phagocytosis, and caveolin-dependent endocytosis [1,2,3]. Moreover, there may be uncharacterized endocytic pathways. Solutes that do not bind to specific receptors may also be internalized through these pathways, along with extracellular fluid via bulk flow. Cargo can be internalized through different endocytic pathways depending on its size [3,4,5]. Although pharmacological inhibitors of these pathways are not specific, cargo smaller than ~200 nm can be internalized through CME, FEME, caveolin-dependent endocytosis, or CLIC/GEEC pathways. Cargo of ~250–500 nm can be internalized by macropinocytosis or phagocytosis. Large cargo, such as bacteria or nanoparticles larger than ~500 nm, is typically internalized by phagocytosis, in which the space between the membrane and cargo is relatively small, whereas macropinocytosis involves large membrane ruffles that enclose extracellular fluid with a larger space between the membrane and cargo [6,7]. Through these endocytic pathways, most cargo is encapsulated by transport carriers, such as vesicles, tubules, macropinosomes, or phagosomes, that subsequently fuse with early endosomes and mature into lysosomes [8,9].

Early endosomes act as sorting hubs that direct cargoes to their respective destinations. Endosomal maturation has been extensively reviewed elsewhere [8,9,10,11,12,13]. Following internalization, early endosomes mature into late endosomes/multi-vesicular bodies (MVBs) by inward budding of the limiting membrane to produce intraluminal vesicles (ILVs) and subsequently into lysosomes for degradation. The bulk flow route is considered to represent the degradation pathway to the lysosomes as early endosomes mature into late endosomes, rather than delivering the cargo to pre-existing late endosomes [12,14]. Simultaneously, specific cargoes are sorted within early endosomes and transported to recycling endosomes for return to the plasma membrane or through retrograde transport pathways to the Golgi apparatus. Cargo that reaches the Golgi apparatus is either recycled back to endosomes or further transported retrogradely to the endoplasmic reticulum.

Along the endocytic pathway, early endosomes are acidified to a pH of 6.2–6.8, followed by further acidification to pH 5.2–6.0 in late endosomes and finally to pH 4.5–4.8 in lysosomes, where the proteins are degraded [15,16,17,18]. Sensitivity to acidic pH has frequently been incorporated into the design of drug delivery systems (DDSs), as endosomal escape is less toxic than permeabilization of the plasma membrane. *Listeria monocytogenes* (*L. monocytogenes*) produces a pore-forming toxin (PFT), listeriolysin O (LLO), whose activity is dependent on acidic pH [19,20]. LLO promotes greater bacterial growth in the cytosol compared with the pH-independent PFT, perfringolysin O (PFO) [21,22], suggesting that the endosomal escape of bacteria into the cytosol is less toxic to host cells compared with entry into the cytosol via the plasma membrane. Consequently, pH-sensitive lipids, polymers, or peptides are incorporated into DDS design as a trigger for drug release from endosomes [21,22,23,24]; however, the efficiency of endosomal escape is very low, even with pH-sensitive, ionizable lipid nanoparticles (1–2% in total cellular siRNA) [25], or pH-sensitive viral peptides (0.17% in antisense RNA delivered to the liver) [26].

In this review, we summarize recent advances in molecular sensors of membrane damage in the plasma membrane, lysosomes and bacteria-containing vacuoles (BCVs) to understand endosomal damage and major methods to damage endosomes. These studies provide insight into molecular sensors that can detect “sterile” endosomal damage induced by DDSs or physical energy-based approaches in the absence of bacterial infection.

There are several differences in endosomal damage induced by DDSs or other artificial methods compared with physiological membrane damage. Bacteria secrete biological factors that regulate host cell activities for their phagosomal escape [27]; however, artificial methods do not secrete such factors. Although lysosomal damage releases highly acidic molecules into the cytosol, the contents released from damaged endosomes may be less acidic compared with those from lysosomes. Damage to the plasma membrane can be highly toxic to cells, whereas damage to the endosomal membrane may be less detrimental.

In this review, we specifically focused on molecules that can immediately detect membrane damage following damage treatment. The early phase of membrane damage detection is often involved in membrane repair and ultimately triggers autophagy to eliminate damaged organelles. Although we describe the autophagosome marker LC3 and ubiquitin in the text, autophagy is a relatively late event and has been extensively reviewed elsewhere [28,29]. Readers may refer to these reviews for a more detailed discussion of organelle degradation mechanisms.

## 2. Molecular Sensors of Membrane Damage in Mammalian Cells

### 2.1. Galectins

#### 2.1.1. Galectin-3 (Gal-3) Is Recruited to Bacteria-Containing Vacuoles and Induces Autophagy

Galectins are glycan-binding proteins that are recruited to damaged membranes, which expose luminal glycans to the cytosolic side [30,31]. Galectin-3 (Gal-3) preferentially binds to internal Galβ(1-3/4)GlcNAc in the glycosylation chain of proteins or glycolipids present on the luminal side of organelles [30,31]. *Shigella flexneri* (*S. flexneri*) is a Gram-negative bacterium that escapes from bacteria-containing vacuoles (BCVs) for proliferation in the cytosol [32,33]. After infection with *S. flexneri* for 30 min, Gal-3 is recruited to the phagosomal membrane surrounding the bacterium in HeLa cells, J774 macrophages, or CHO cells [34,35]. Gal-3 is recruited to tubular membranes connected to the limiting membrane of BCVs instead of the bacterium itself [35]. The glycan-binding ability of Gal-3 is required for this localization; therefore, Gal-3 recognizes glycans exposed to the cytosol by membrane damage occurring in the inner leaflet of host BCV membranes [35].

Gal-3 directs damaged organelles to the autophagy pathway for degradation [36,37]. Autophagy is a process in which cytosolic components are sequestered and degraded within double-membrane structures marked by lipidated LC3 [28,38]. After internalizing *S. flexneri,* Gal-3 colocalizes with ubiquitin, p62, and LC3 into damaged BCVs [34]. p62 is an autophagy adaptor that binds polyubiquitin chains and links ubiquitinated cargo to LC3 for autophagy [39,40]. Consistent with these observations, Gal-3 KO mice show increased susceptibility to infection by *Mycobacterium tuberculosis* (*Mtb*) [36]. These results indicate that Gal-3 plays a role in host cell defense against bacterial infections.

Although ubiquitination of escaped bacteria in the cytosol plays an important role in triggering antibacterial autophagy, ubiquitination and recruitment of E2 and E3 ligases to bacteria occur later than Gal-3. Gal-3 recruitment is detectable within 5–30 min after infection. On the other hand, *S. flexneri* was directly ubiquitinated on its lipopolysaccharide (LPS) by the host E3 ligase RNF213 and was detectable 2 h post-infection [41,42]. In *Mtb*, bacterial ubiquitination is mediated by E3 ligases, in which Parkin is detectable within ~4 h and Smad ubiquitination regulatory factor 1 (Smurf1) is detectable within ~15 h following infection [43,44]. In addition, the E3 ligase leucine-rich repeat and sterile alpha motif-containing protein 1 (LRSAM1) is recruited to internalized *Salmonella enterica serovar Typhimurium* (*S. Typhimurium*) and *S. flexneri* and is detectable within ~45 min [45]. Taken together, these results indicate that Gal-3 detects damaged BCVs, rather than bacteria themselves, as an earlier event than bacterial ubiquitination during infection.

#### 2.1.2. Gal-3 Is Recruited to “Sterile” Lysosomal Membrane Damage and Induces Autophagy

Gal-3 is recruited to the “sterile” damage of phagosomes induced by internalization of 3 μm latex beads coated with cationic lipid transfection reagents [46], as well as to damaged lysosomes by internalization of silica nanoparticles or treatment of L-leucyl-L-leucine methyl ester (LLOMe) [47]. LLOMe destabilizes the lysosomal lipid bilayer by forming a polymer from a leucine dipeptide in a cathepsin C-dependent manner [48]. Therefore, it is often used as a lysosome-specific damaging agent. In live imaging, Gal-3 recruitment was observed in damaged lysosomes within 5 min [49]. These results indicate that Gal-3 recognizes glycan-exposed, damaged organelle membranes and does not require bacterial infection.

Treatment with LLOMe recruits Gal-3 to damaged lysosomes, where it colocalizes with ubiquitin, LC3, and ATG16L, which direct ubiquitinated lysosomes for the autophagy pathway [46,50]. Gal-3 interacts with tripartite motif-containing protein 16 (TRIM16), which in turn recruits ATG16L to damaged lysosomes [36]. Gal-3 depletion partially decreases LC3 recruitment following LLOMe treatment [37], suggesting that it is involved in recruiting LC3. LC3 and most other autophagy components are recruited 5–20 min after the recruitment of Gal-3 to damaged phagosomes [46]; therefore, most of the autophagy machinery is recruited to damaged lysosomes later than Gal-3. However, ubiquitin and p62 are recruited to damaged endosomes/lysosomes concomitantly with Gal-3 [46]. Ubiquitin-K63 recruitment was detected even before Gal-3 upon LLOMe treatment [51]. Therefore, ubiquitin and p62 recruitment may occur in parallel or before Gal-3 recruitment.

Screening of E2 ubiquitin-conjugating enzymes following LLOMe treatment was conducted, and UBE2QL1 was identified [52]. UBE2QL1 is translocated to lysosomes 30–60 min after LLOMe treatment, but not to damaged mitochondria [52]. Depletion of UBE2QL1 abolishes ubiquitin signals as well as the recruitment of LC3, suggesting that UBE2QL1 is specifically required for directing damaged lysosomes for degradation [52,53]. Gal-3 recruitment to damaged lysosomes is enhanced following UBE2QL1 depletion, suggesting that Gal-3 is recruited to the damaged membrane independently of ubiquitin and can trigger autophagy.

#### 2.1.3. Gal-3 Promotes the Repair Pathway of Damaged Lysosomes

Gal-3 also plays a role in membrane repair. It binds to ALIX, which is an endosomal sorting complex required for transport (ESCRT) protein [54] (Section 2.2.5). ALIX and ESCRT proteins are involved in membrane repair. Although Gal-3 is not important for ALIX recruitment to damaged lysosomes during the first 10 min following LLOMe treatment, ALIX recruitment is dependent on Gal-3 after 30 min [37]. ALIX depletion reduces lysosomal recovery even after 30 min of LLOMe washout [37], suggesting that ALIX-dependent membrane repair via Gal-3 is important at the later stages of membrane damage. These results support the idea that following membrane damage, ALIX and ESCRT machinery function at the initial stage for membrane repair. Afterwards, while Gal-3 maintains the membrane repair pathway by binding to ALIX, Gal-3 triggers the autophagy pathway [37]. Interestingly, Gal-3 recruitment is independent of Ca^2+^ [55]. As Ca^2+^ influx functions during the first-line response to other proteins, Gal-3 could be the second-line sensing molecule that detects membrane damage when the Ca^2+^-dependent process is unavailable.

#### 2.1.4. Galectin-8 (Gal-8) Detects Larger Rupture of Membrane Damage Related to Pathogens

Galectin-8 (Gal-8), another galectin family protein, binds to Galβ(1-3)GalNAc, and its conjugation to sialic acid or fucose increases its affinity for Gal-8 [56]. Gal-8 also binds to Galβ(1-4)GlcNAc with lower affinity [31,56]. After infection with *S. Typhimurium* in HeLa cells, of the 11 galectins tested, only Gal-3, -8, and -9 were recruited to Salmonella-containing vacuoles (SCVs), which are unique phagosomes that *S. Typhimurium* forms without escaping to the cytosol [57]. Of these galectins, only the depletion of Gal-8 increased the proliferation of *S. Typhimurium* [57], suggesting that Gal-3 and Gal-9 are not directly involved in inhibiting *S. Typhimurium* proliferation. Gal-8 preferentially interacts with the autophagy adaptor NDP-52, rather than p62 [38]. Consistently, NDP-52 depletion increased *S. Typhimurium* proliferation [57], which supports the roles of Gal-8 and NDP-52 in antibacterial activity against *S. Typhimurium.* Gal-8 requires 10–15 min for SCV recruitment [58] and detects a relatively larger rupture of membrane damage (Section 2.3.2). More Gal-8 is recruited to adenovirus-damaged endosomes compared with LLOMe-damaged lysosomes [59,60]. Therefore, Gal-8 appears to detect more pathogen-related membrane damage than sterile membrane damage.

#### 2.1.5. Galectin-9

Galectin-9 (Gal-9), like Gal-3, binds to internal Galβ(1-3/4)GlcNAc residues [30,31]. It is required for ubiquitination in damaged lysosomes induced by LLOMe [61]. Depletion or mutation of Gal-9, resulting in a lack of ability to bind to glycosylated chains, decreases ubiquitination in damaged lysosomes induced by LLOMe. Gal-9 binds to the deubiquitinating enzyme USP9X, and its recruitment sequesters USP9X, resulting in more ubiquitination of damaged lysosomes. Gal-9 also excludes USP9X from TAK1, which results in the ubiquitination and phosphorylation of TAK1. TAK1 phosphorylates AMP-activated protein kinase (AMPK), which triggers autophagy [62,63]. Consistently, Gal-9 depletion inhibits LC3 lipidation following LLOMe treatment [61]. Although Gal-9 is involved in immune function [64,65], it has been used as a readout of endosomal membrane damage to identify efficient lipid nanoparticle formulations because of its rapid and more efficient ability to detect damage to endosomes [66] (Section 3.1 and Section 3.5).

### 2.2. ESCRTs

#### 2.2.1. Overview of the ESCRT Proteins

Endosomal sorting complexes required for transport (ESCRT) proteins are class E vacuolar protein sorting (Vps) proteins and include ESCRT-0, I, II, III, and the Vps4 ATPase [67,68,69,70,71,72]. Studies on ESCRT proteins in the trafficking of the epidermal growth factor receptor (EGFR), a well-known receptor tyrosine kinase [73,74,75], have increased our understanding of the roles of ESCRT complexes. Following EGF binding, EGFR is ubiquitinated [76,77,78,79,80], and Hrs and STAM complexes and ESCRT-0 components bind to ubiquitinated EGFR and transfer it to ESCRT-I, II, and III to induce inward budding and produce ILVs [11,81,82]. Through inward budding, the phosphorylated cytosolic region of EGFR, which binds signaling complexes such as Grb2 [83,84], is incorporated into ILVs and degraded in lysosomes along with the luminal region of EGFR [74,75]. CHMP4A and CHMP4B, which are ESCRT-III proteins, form filaments [85], and ESCRT-III mediates membrane scission to form ILVs [86,87]. ESCRT-III assembles at the bud neck and constricts the membrane along with Vps4 to sever the remaining membrane [88,89]. Thus, ESCRT proteins form vesicles by pushing the membrane from the cytosol into the lumen. This is an inverse mechanism compared with coat proteins, such as clathrin or COPI, which invaginate vesicles by pulling the membrane from the lumen to the cytosol. ESCRT proteins have many functions beyond ILV formation, which have been reviewed elsewhere [82].

#### 2.2.2. ESCRT in Plasma Membrane Damage

ESCRT machinery plays an important role in membrane repair. Plasma membranes can be damaged by digitonin, streptolysin O, or UV laser ablation, and CHMP4B, an ESCRT-III component, is recruited to the wound site [90]. CHMP4B is detectable within 1 min following plasma membrane damage by a UV laser. CHMP4B depletion decreases cell survival following wounding, indicating that the ESCRT machinery is important for plasma membrane repair [90]. Based on the kinetics of entry of propidium iodide (PI) to the nucleus from the plasma membrane lesion, depletion of ESCRT subunits, such as CHMP4B and Vps4, is especially deleterious for cells containing less than 100 nm wounds [90]. As CHMP4B-positive blebs are produced at wounded sites on the plasma membrane, ESCRT-III and Vps4 may constrict intact membrane regions to shed damaged membrane portions as extracellular vesicles for membrane repair [90].

#### 2.2.3. Ca^2+^ Release Recruits ALG2 and ALIX to Plasma Membrane Lesions Within 30 s

The ESCRT subunits involved in plasma membrane damage repair are not identical to those functioning in EGFR sorting into ILVs. Although ALG2, TSG101, ALIX, and CHMP4B are recruited to the plasma membrane by Ca^2+^, the ESCRT-0 components Hrs and STAM, the ESCRT-I subunit Vps37, and the ESCRT-II subunit Vps25 are not detected [90,91].

Apoptosis-linked gene 2 (ALG2) binds to ALG-2 interacting protein 1 or X (AIP1/ALIX) in a Ca^2+^-dependent manner [92,93,94,95] ALIX binds to TSG101 and CHMP4B to bridge Tsg101/ESCRT-I and ESCRT-III [96,97,98,99]. Notably, upon plasma membrane injury, ALG2 and ALIX are recruited to plasma membrane lesions within 30 s, followed by CHMP4B and Vps4B within 40–55 s [90,91]. Depletion of ALG2 disrupts the recruitment of ALIX, CHMP4B, and Vps4B to the plasma membrane [91], suggesting that the Ca^2+^-dependent binding of ALG2 to the damaged membrane is necessary for subsequent recruitment of ESCRT components.

ALG2 binds to another Ca^2+^-dependent protein, annexin A7, which may function even earlier than ALG2 [100] (Section 2.7.2). Other Ca^2+^-dependent proteins, including IQGAP1, have been implicated in plasma membrane repair with ATG9 (Section 2.6.2). Taken together, Ca^2+^ release into the cytosol induces multiple pathways that are dependent or independent of ESCRT machinery to initiate plasma membrane repair.

Ubiquitin is also observed at the damaged plasma membrane. Although CHMP4B is detected 10–60 s following injury, ubiquitination is only evident within 6 min [90]. Therefore, ubiquitin conjugation may occur after the initial membrane repair process.

#### 2.2.4. ESCRT Is Recruited to Smaller Membrane Damage of Lysosomes Dependent on Ca^2+^

ESCRT proteins are also involved in the repair of damaged lysosomal membranes, as in plasma membrane damage [55,101]. The lysosome-damaging agents LLOMe and glycyl-L-phenylalanine 2-naphthylamide (GPN), a substrate of cathepsin C that induces osmotic lysis of lysosomes [102,103,104], recruit ALIX and CHMP4B to damaged lysosomes [37,55,101]. ALIX and TSG101 depletion disrupts CHMP4B recruitment and prevents recovery of lysosomal pH [37,55,101], suggesting that the ESCRT complex is required for lysosomal repair.

Ca^2+^ is also involved in the recruitment of the ESCRT complex to damaged lysosomes. The Ca^2+^ chelator, BAPTA-AM, inhibits CHMP4B recruitment to damaged lysosomes [55,105], and exacerbates lysosomal damage [105]. ALG2 depletion exacerbates lysosomal damage [105], which suggests that ALG2 is important for lysosomal repair. Lysosomal damage induced by GPN increases juxta-lysosomal Ca^2+^. The addition of ML-SA5, an agonist of the lysosomal Ca^2+^ channel TRPML1, exerts protective effects on lysosomal damage induced by GPN [105]. Therefore, Ca^2+^ plays an important role in lysosomal repair.

The ESCRT machinery is preferentially recruited to small membrane lesions of damaged lysosomes earlier than Gal-3 and annexins A1 and A2. ESCRT is recruited to damaged lysosomes that retain 40 kDa or 10 kDa dextran after LLOMe treatment [55]. In contrast, Gal-3 is recruited only after the release of 40 kDa dextran [55], whereas annexins A1 and A2 are recruited after the release of 10 kDa dextran [106] (Section 2.7.3). These findings support the idea that the ESCRT machinery detects smaller membrane lesions than Gal-3 and annexins A1 and A2.

#### 2.2.5. Ca^2+^-Dependent and Independent Sensing of Lysosomal Membrane Damage

SPG20 binds to IST1 and is recruited to damaged lysosomes 10 min after LLOMe treatment [51]. IST1 is an ESCRT-III-like protein that binds to ALG-2 in a Ca^2+^-dependent manner to form a complex with CHMP1A and CHMP1B [51,107]. IST1 is recruited to the lysosomes 60 s after LLOMe treatment [51] and 2 min after GPN treatment [105]. IST1 linking ALG2 to ESCRT-III is dependent on Ca^2+^.

Deletion of the IST1-binding domain in SPG20 only partially reduces SPG20 recruitment to damaged lysosomes [51]. SPG20 contains an SC domain that binds to loosely packed lipids generated by lipid peroxidation. Lipid peroxidation induces SPG20 recruitment to damaged lysosomes, whereas Ca^2+^ release without lipid-packing defects, induced by ML-SA1, an agonist of the lysosomal Ca^2+^ channel TRPML1, does not recruit SPG20, even though IST1 can be recruited under these conditions [51]. These results indicate that SPG20 recognizes damaged membranes independent of Ca^2+^, in addition to being bound to IST1 [51,107].

SPG20 forms a complex with the ubiquitin ligase ITCH, which is also recruited to damaged lysosomes in 15 min [51]. ITCH depletion reduces ubiquitination on lysosomes, reduces LC3 recruitment to damaged lysosomes, and prevents the clearance of Gal-3-positive damaged lysosomes by lysophagy [51]. These results indicate that SPG20 senses defects in lipid packing, which leads to lysosomal ubiquitination by ITCH and induces clearance of damaged lysosomes by lysophagy. Although the Ca^2+^-dependent repair process begins within the first 60 s after lysosomal damage, lipid damage detection and the subsequent lysophagy process begin after approximately 10 min. These findings support a model in which Ca^2+^ serves as the initial signal for lysosomal membrane damage, triggering IST1-dependent repair, followed by SPG20-mediated detection of lipid-packing defects.

Although the Ca^2+^-dependent repair process begins during the first 60 s after lysosomal damage, detecting lipid damage and the subsequent lysophagy process begin after 10 min, thus supporting the sequence that Ca^2+^ is the early signal of lysosomal membrane damage dependent on IST1, followed by SPG20 detecting lipid-packing defects independent of Ca^2+^.

#### 2.2.6. ESCRT in Bacteria-Containing Vacuoles

Depletion of CHMP3, an ESCRT-III component, induces the enlargement of *Salmonella*-containing vacuoles (SCVs) and significantly increases the cytosolic proliferation of *S. Typhimurium* [108]. This suggests that ESCRT proteins play important roles in controlling the size and morphology of SCVs and restricting bacterial proliferation. *Coxiella burnetii*, which is a Gram-negative bacterium that proliferates within lysosomes, also induces ESCRT recruitment to the lysosomes [55,101].

The deubiquitination enzyme USP8 regulates the balance between ESCRT and autophagy. USP8 depletion enhances ubiquitination around infected *Mtb* and restricts *Mtb* proliferation [109]. Conversely, USP8 also removes ubiquitin from ESCRT-III components, such as CHMP2B and CHMP4 [110]. USP8 depletion increases the levels of CHMP4B at damaged lysosomes induced by LLOMe [109]; however, Vps4 recruitment is disrupted in USP8-depleted cells [109], suggesting that deubiquitination of CHMP4B is required for Vps4 recruitment to accomplish the repair process. USP8 depletion enhances the recruitment of autophagy receptors, such as SQSTM1/p62 [109]. These findings support the idea that enhanced ubiquitination in damaged lysosomes attenuates ESCRT-mediated membrane repair and promotes autophagy.

### 2.3. Sphingomyelin

#### 2.3.1. Overview of Sphingomyelin and Its Detection

Sphingomyelin is synthesized from ceramide in the lumen of the Golgi apparatus [111]. It is subsequently transported to the plasma membrane, endosomes, and lysosomes [112,113,114,115]. It is predominantly localized to the luminal leaflet of intracellular organelles or the outer leaflet of the plasma membrane [112]; however, it is exposed to cytosolic leaflets in the lipid bilayer of damaged organelles [49,58].

Sphingomyelin can be detected by the earthworm toxin lysenin, the C-terminal region of lysenin, a sphingomyelin-binding proteinaceous toxin isolated from the earthworm *Eisenia foetida* [116], as well as by equinatoxin II (EqtII), a PFT isolated from *Actinia equina* [117]. Under normal conditions, sphingomyelin is localized to the lumen of organelles [118,119]. Lysenin and EqtII do not completely colocalize [119]. In the presence of glycolipids that are miscible with sphingomyelin, the binding of lysenin to sphingomyelin decreases [120], indicating that lysenin binds to highly clustered sphingomyelin, but not to sphingomyelin mixed with glycolipids. Lysenin stained large puncta in the plasma membrane as well as sphingomyelin in late endosomes but not in the Golgi apparatus, early endosomes, or recycling endosomes [119]. In contrast, EqtII detects sphingomyelin in smaller puncta in the plasma membrane, late endosomes, and recycling endosomes [119]. EqtII binds to sphingomyelin dispersed in glycolipids; thus, EqtII can detect more sphingomyelin in diverse organelles. EqtSM represents a modified EqtII domain without a signal peptide and can be expressed in the cytosol to detect cytosol-exposed sphingomyelin [49].

#### 2.3.2. Sphingomyelin Is Exposed in Smaller Rupture of Membrane Damage in BCVs

BCV membrane damage induced by *S. Typhimurium*, *S. flexneri*, *L. monocytogenes*, and *Streptococcus pyogenes* infection exposes sphingomyelin to the cytosolic leaflet of the lipid bilayer, detected by lysenin [58]. Lysenin recruitment was inhibited by the expression of neutral sphingomyelinase 2 (nSMase2), which hydrolyzes sphingomyelin on the cytosolic side, suggesting that it is present in the cytosolic leaflet. Recruitment of lysenin to BCVs precedes that of Gal-8. Electron microscopy revealed that damaged membranes positive only for lysenin contain gaps of approximately 100–200 nm, whereas membranes positive for lysenin and Gal-8 exhibit larger discontinuities and no longer enclose *S. Typhimurium*. These results suggest that exposure of sphingomyelin to the cytosol occurs at an earlier stage compared with Gal-8 recruitment during phagosomal membrane damage [58]; however, nSMase expression does not inhibit Gal-8 recruitment to SCVs [121], suggesting that Gal-8 is recruited independently of sphingomyelin.

Tectonin domain-containing protein 1 (TECPR1) is an endogenous receptor that binds to sphingomyelin exposed to the cytosol [121]. It binds ATG5, and its expression promotes the recruitment of LC3 and ATG5 to autophagosomes containing *Shigella ΔicsB* [122], which are unable to escape from BCVs [123]. TECPR1 depletion prevents the recruitment of LC3 to the *Shigella ΔicsB*-positive autophagosomes and fails to suppress the multiplication of *Shigella ΔicsB* in HeLa cells [122]; however, TECPR1 depletion does not inhibit LC3 recruitment to SCVs in mouse embryonic fibroblast cells [121]. Although ATG5 recruitment to SCVs is inhibited in TECPR1-depleted cells, the major pathway for LC3 recruitment is dependent on ATG16L in this case [121]. These results suggest that TECPR1 is involved in autophagy.

#### 2.3.3. Sphingomyelin Exposure in the Damaged Plasma Membrane

Sphingomyelin is exposed to the cytosol in damaged plasma membranes [49]. EqtSM is recruited to the damaged plasma membrane within 5 s following laser-induced injury [49]. Ca^2+^ chelation by EGTA or BAPTA-AM significantly reduces EqtSM recruitment to the damaged plasma membrane following treatment with streptolysin O (SLO), a PFT from *Streptococcus pyogenes* [49]. Depletion of TMEM16F, a calcium-activated phospholipid scramblase localized to the plasma membrane, abolished EqtSM recruitment to the SLO-damaged plasma membrane. This indicates that TMEM16F scrambles sphingomyelin from luminal to cytosolic leaflets, which is dependent on Ca^2+^ at the plasma membrane.

#### 2.3.4. Sphingomyelin Exposure in Lysosomal Membrane Damage

Lysenin is recruited to damaged lysosomes within 15 min [58], and EqtSM is also recruited within 5 min following LLOMe treatment [49], suggesting that sphingomyelin is also exposed to the cytosolic side of damaged lysosomes. Ca^2+^ chelation by EGTA or BAPTA-AM reduces EqtSM recruitment to the damaged lysosomes; therefore, Ca^2+^ plays an important role in sphingomyelin exposure [49]; however, depletion of TMEM16F, the plasma membrane phospholipid scramblase, did not affect EqtSM recruitment to LLOMe-damaged lysosomes [49]. Therefore, an alternative Ca^2+^-dependent scramblase may be involved in the exposure of sphingomyelin in damaged lysosomes. Sphingomyelin synthase SMS1 and SMS2 depletion attenuates the recovery of lysosomal acidification following the removal of GPN; therefore, sphingomyelin is essential for lysosomal repair. Although nSMase removes phosphocholine from sphingomyelin to generate ceramide [124,125,126], nSMase activity promotes lysosomal recovery following GPN removal [49]. GW4869, an nSMase inhibitor, hinders the recovery of lysosomal acidification after GPN removal. Therefore, upon lysosomal damage, sphingomyelin is translocated to the cytosolic leaflet and digested by nSMase to generate ceramide, which could bend the membrane to repair the damaged membrane [49].

Depletion of ESCRT components, including CHMP3, ALIX, and TSG101, did not inhibit EqtSM recruitment [49]. Moreover, depletion of sphingomyelin synthase and ALIX/TSG101 had additive effects on cell survival, and nSMase activity became more important for lysosomal recovery in ALIX/TSG101-depleted cells. These results suggest that sphingomyelin-dependent responses are independent of the ESCRT pathway [49].

TECPR1 detects cytosol-exposed sphingomyelin in damaged lysosomes induced by LLOMe [121]. TECPR1-positive structures are often positive for Gal-8. However, there are also Gal-8-negative, TECPR1-positive structures [121]. These structures may exhibit less damage; sphingomyelin is exposed but still maintains the integrity of lysosomes, and intralumenal glycans are not exposed. TECPR1 could detect smaller lesions, similar to those detected by lysenin [58,121]. TECPR1 induces the autophagy pathway in lysosomal damage [127].

### 2.4. Stress Granules

Proteomic approaches have indicated that damaged lysosomes associate with stress granule components, G3BP1 and NUFIP2 [128]. G3BP1 puncta formation is induced by lysosomal damage following LLOMe treatment, GPN treatment or silica internalization. G3BP1 puncta become detectable after 3 min of LLOMe treatment in live imaging and dynamically associate with damaged lysosomes [129]. Localization of G3BP1 and Gal-3 in damaged lysosomes is juxtaposed rather than colocalized, suggesting that stress granules plug damaged lysosomes. G3BP1 and G3BP2 depletion delays the recovery of lysosomal acidification after LLOMe washout [129]. Therefore, stress granule formation facilitates lysosomal repair.

G3BP1 and G3BP2 depletions increased the replication of *M. tuberculosis*, suggesting that stress granules play a role in restricting bacterial proliferation in host cells [129]. Stress granule formation may also be induced by various pathogenic lysosomal damage, including adenovirus infection, SARS-CoV-2 ORF3a protein, malarial pigment hemozoin, silica crystals, and tau aggregates [128,130], indicating the importance of stress granule formation in modulating the effects of lysosomal damage.

How stress granules are formed upon lysosomal damage has been examined. G3BP1 forms condensates in damaged artificial vesicles with poly(A)-RNA at low pH; therefore, mixing lower osmolarity and low pH solutions with RNA may result in condensation of G3BP1 [129]. Another report indicated that Ca^2+^ release from lysosomes is accompanied by phosphorylation of eIF2α and inhibits global translation, which results in the accumulation of untranslated mRNAs. A search for proteins that bind to eIF2α under LLOMe treatment identified PKR and its activator PACT as upstream kinases upon lysosomal damage to eIF2α [128,130]. ALIX binds to PKR and PACT during LLOMe treatment, and depletion of Ca^2+^ or ALIX inhibits eIF2α phosphorylation. Therefore, Ca^2+^ leakage and recruitment of ALIX/ALG2 to damaged lysosomes may lead to eIF2α phosphorylation, resulting in stress granule formation.

Stress granules can form independently of the autophagy machinery. NUFIP2, a stress granule protein, binds to GABARAP, a member of the LC3-like mammalian ATG8 (mATG8) family, and is recruited to the autophagic membrane [128]. Although mTOR is normally released from lysosomes following LLOMe treatment, NUFIP2 depletion prevents the dissociation of mTOR from damaged lysosomes [128], suggesting that NUFIP2 contributes to the regulation of mTOR signaling. In contrast, the depletion of GABALAP and other mATG8 proteins results in increased stress granule formation, though it prevents mTOR dissociation from damaged lysosomes [128]. Similarly, Gal-3 depletion enhances stress granule formation [130]. These results indicate that stress granule formation occurs independently of both autophagy and Gal-3 pathways and protects cells from the cytotoxic effects of lysosomal damage [130].

### 2.5. PI4P, ORPs and ATG9 in Membrane Contact Sites for Membrane Repair

#### 2.5.1. Overview of Membrane Contact Sites

Membrane contact sites (MCSs) form close contact between the membranes of different organelles [131,132,133]. MCSs often mediate lipid transfer between different organelles by lipid transfer proteins (LTPs) [132,133]. Oxysterol-binding protein (OSBP) and OSBP-related proteins (ORPs) are LTPs that are frequently localized in MCSs at endosomes, lysosomes, and the Golgi apparatus. In this section, we describe ORP9, ORP10, ORP11, ORP1L, and OSBP. Of these, ORP9, ORP1L, and OSBP contain an FFAT motif that binds to VAP-A and VAP-B localized to the endoplasmic reticulum (ER) and forms MCSs between the ER and endosomes, lysosomes, or the Golgi apparatus [132,133,134]. Although ORP10 and 11 do not have an FFAT motif, they form heterodimers with ORP9 and form MCSs [134,135,136]. ORP9, 10, 11 and OSBP bind to phosphatidylinositol-4 phosphate (PI4P), which is dependent on their pleckstrin homology (PH) domain. On the other hand, their ORD domains also bind PI4P in the post-Golgi membranes and exchange PI4P for phosphatidylserine (PS) (ORP)/10/11) or cholesterol (OSBP) from the ER [134,135,136,137,138]. Consequently, PS or cholesterol is transferred from the ER to the post-Golgi membranes, whereas PI4P transferred to the ER is hydrolyzed by the phosphoinositide phosphatase Sac1 [133].

PI4P may be visualized using PI4P-binding probes, such as the PH domain of OSBP or FAPP1 [139]; however, the localization of OSBP-PH to the Golgi depends on PI4P and Arf1, a small GTP-binding protein essential for vesicle budding at the Golgi [140]. Inhibition of Arf1 localization at the Golgi also disrupts the Golgi localization of OSBP-PH [139], indicating that Golgi targeting of OSBP-PH requires PI4P and Arf1. As a result, PI4P-specific probes have been developed. The P4M domain of SidM from *Legionella pneumophila* detects PI4P, which localizes to the Golgi apparatus as well as the plasma membrane, late endosomes, and lysosomes but less in early endosomes [141].

#### 2.5.2. PI4P in Lysosomal Damage

Proteomic and lipidomic analyses of damaged lysosomes indicate that the lipid transfer pathway is increased upon lysosomal injury [137,142]. The PI4P probe, SidM, is recruited to damaged lysosomes 5–10 min after LLOMe treatment [142], and OSBP-PH is also recruited to damaged lysosomes within 30 min [137]. Phosphatidylinositol 4-kinase type IIα (PI4KIIA) depletion abolishes OSBP-PH recruitment to damaged lysosomes, suggesting that PI4P production at damaged lysosomes requires PI4KIIA [137]. PI4KIIA is recruited to damaged lysosomes approximately 10 min after LLOMe treatment [137]. Importantly, activation of the lysosomal Ca^2+^ channel TRPML1 by its agonist ML-SA1 is sufficient to trigger PI4KIIA recruitment, suggesting that Ca^2+^ leakage is a key signal for the recruitment of PI4KIIA to damaged lysosomes [137]. ALIX and TSG101 depletion does not affect PI4P production in damaged lysosomes, and conversely, PI4KIIA depletion does not hinder the recruitment of ALIX, CHMP4B, and Gal-3 to damaged lysosomes, suggesting that the PI4KIIA pathway is independent of the ESCRT machinery and Gal-3 [142].

Within 10 min after LLOMe treatment, ORP1L, ORP9, 10, 11, and OSBP are recruited to damaged lysosomes [137,142]. LLOMe treatment extensively increased ER–lysosome MCSs [137,142]. Although ORP10 and 11 do not have an FFAT motif, depletion of VAP-A/B attenuates the formation of ER–lysosome MCSs [142]. Similarly, ORP9, 10, 11, and OSBP quadruple knockout decreased VAP-A clustering around damaged lysosomes [137], suggesting that VAP-A/B and ORP9/10/11 and OSBP form MCSs in lysosomes upon lysosomal damage.

ORP9 and 11 transfer PS to PI4P liposomes in vitro, and LLOMe treatment increased PS in lysosomes [137]. PS accumulation is rescued by any two ORPs, ORP9/10, 9/11, or 10/11, and reduces Gal-3 recruitment to the damaged lysosomes. These results support the hypothesis that PS transfer to damaged lysosomes plays an important role in lysosomal repair.

Cholesterol also plays a role in repairing damaged lysosomes. OSBP transfers cholesterol, and OSBP depletion reduces cell survival after LLOMe treatment. Accumulation of cholesterol in lysosomes decreases Gal-3 recruitment to damaged lysosomes, indicating that cholesterol protects lysosomes from damage similarly to PS [142].

Apilimod inhibits the production of PI(3,5)P2 from PI3P, accumulates PI4P in the lysosomes, and enhances their repair [143]. PI(3,5)P2 is required for the invagination of lysosomes for microautophagy [144]; however, lysosomal acidification is preserved, suggesting that lysosomal integrity is not overtly perturbed [143]. Inhibiting the PI3P-to-PI(3,5)P_2_ conversion or inhibiting PI4KIIA enhances the exocytic disposal of the contents of MVBs and lysosomes [145,146]. These results suggest that mild damage to lysosomes induces MVB or lysosomal secretion, an additional response to lysosomal damage.

### 2.6. ATG9 for Membrane Repair

#### 2.6.1. Overview of ATG9

ATG9 is an essential protein for autophagy [147,148]. It is a multimembrane-spanning protein that cycles between the TGN and late and recycling endosomes [149,150]. When autophagy is activated, ATG9 relocalizes to precursors of the autophagosome [150,151]. ATG9 forms a homotrimer complex [152] and possesses lipid scramblase activity required for autophagosome formation [153,154].

ATG9 binds to ATG2 [155]. ATG2 was discovered as an essential component of autophagy in yeast [28,29,156,157]. It possesses a cavity that binds to lipids and exhibits lipid transfer activity [158,159,160]. Similarly, the human homologs ATG2A and 2B bind to phospholipids and have lipid transfer activity in vitro [160,161]. Although ATG2 can transfer lipids independently of ATG9 in vitro, efficient membrane expansion of autophagosomes in cells is thought to require ATG9-mediated scramblase activity. ATG2 has been proposed to form a complex with ATG9, in which ATG2 transfers lipids to the membrane, and ATG9 facilitates their distribution across the bilayer through its scramblase activity, thereby promoting membrane expansion [153,154,155,162].

ATG9 binds to many vesicle budding machineries, including clathrin adaptors, AP-2 [163,164], AP-4 [165,166], AP-1 [167], and AP-3 [168], as well as the retromer complex [163]. Upon depletion of AP-2 or AP-4, ATG9 remains in the recycling endosomes or TGN even under starvation conditions, and the autophagy pathway is inhibited, suggesting that the transport of ATG9 from its original organelles to autophagosomes is required for autophagosome formation [163,164,165].

#### 2.6.2. ATG9 in Plasma Membrane Damage

When the plasma membrane is damaged, ATG9 is recruited to the plasma membrane [169,170] within 1 min by digitonin, 5 min by SLO, or 1 min and 30 s by glass bead injury [169]. Plasma membrane permeabilization was exacerbated in ATG9-depleted cells, indicating that ATG9 protects the membrane from damage. ATG9 is recruited to the injured plasma membrane in a manner dependent on IQGAP1, which responds to Ca^2+^. Depletion of Ca^2+^ outside of cells inhibits ATG9 recruitment to the injured plasma membrane [169], which suggests that Ca^2+^ influx from the plasma membrane is important for ATG9 recruitment. IQGAP1 interacts with ATG9 and the ESCRT-III protein, CHMP2A. Double knockout of ATG9 and CHMP2B does not exacerbate PM permeabilization compared with the single knockout of either. These results indicate that ATG9 and CHMP2B function in the same pathway for plasma membrane repair [169]. TSG101 depletion inhibits the recruitment of ATG9 to the damaged plasma membrane, supporting the hypothesis that ATG9 functions downstream of the ESCRT machinery [170].

ATG9 scramblase activity is required for plasma membrane repair [170]. Its mutants lacking scramblase activity are recruited to damaged plasma membranes; however, plasma membrane permeabilization is not restored, as measured by PI flux to the nucleus [170]. In addition, Vps13A, another lipid transfer protein that binds to ATG9 [171], is required for plasma membrane repair by ATG9 [170]. Vps13A is a lipid transfer protein and forms a complex with ATG9, distinct from ATG2 [171]. Therefore, the ATG9-Vps13A complex may specifically supply lipids for plasma membrane repair.

#### 2.6.3. ATG9 in Lysosomal Damage

ATG9 plays an important role in lysosomal repair [168]. In LLOMe-treated cells, ATG9 is translocated from the Golgi area to damaged lysosomes within 15 to 45 min [168]. Depletion of cellular Ca^2+^ inhibits ATG9 recruitment, indicating that Ca^2+^ promotes ATG9 recruitment [168]. ATG9 depletion prevents PI4KIIA localization to the lysosomes, indicating that ATG9 is required for PI4KIIA translocation from the TGN to the lysosomes [143].

ATG2A also localizes to lysosomes following LLOMe treatment in 5–10 min [137]. Depletion of ATG2A/B results in a persistent increase in Gal-3 puncta following LLOMe treatment, suggesting that ATG2A/B is required for lysosomal repair. ATG2A mutants lacking lipid transfer activity also result in more Gal-3 puncta than wild-type ATG2A, indicating that the lipid transfer activity of ATG2A/B contributes to the repair of damaged lysosomes [137]. Although ATG9 has been proposed to form a complex with ATG2 for autophagosome expansion [153,155,162], whether ATG9 and ATG2A/B function together as a single functional complex during lysosomal repair remains unclear.

ATG9 vesicles contain ARFIP2/Arfaptin 2, an Arf-GTP-binding protein [140], and bind to PI4P and PI4PK2A [168,172]. ARFIP2 depletion increases ATG9, PI4KIIA, and PI4P localization to damaged lysosomes, which are repaired faster [168]. ARFIP2 forms a complex with AP-3. When AP-3 is depleted, ATG9 increases in damaged lysosomes, which suggests that AP-3 retrieves ATG9 from the endolysosomal compartment to the Golgi and attenuates lysosomal repair [168]. ARFIP2 binds to ORP9 and interferes with the PS lipid transfer activity of ORP9/11 in vitro [168]. ARFIP2 depletion restricts *Mtb* and *Salmonella* infection, which indicates that ARFIP2 inhibits lysosomal repair under normal conditions, whereas ARFIP2 depletion promotes lysosomal repair [168]. These results indicate that ATG9 plays a role in promoting lysosomal repair by translocating PI4KIIA to damaged lysosomes. In contrast, clathrin adaptors and ARFIP2 restrict the repair process by removing ATG9 from lysosomes.

### 2.7. Annexins

#### 2.7.1. Overview of Annexins

Annexins are a family of Ca^2+^-dependent phospholipid-binding proteins [173,174,175]. They bind to negatively charged phospholipids, including PS, PI, and PI(4,5)P2, in a Ca^2+^-dependent manner [176,177,178,179,180,181,182]. Of these, PS is primarily used for Ca^2+^-dependent binding of annexins to lipids. Although annexins bind to lipids primarily dependent on Ca^2+^, annexin A2 can associate with early endosomes in a cholesterol-dependent but Ca^2+^-independent manner [183,184].

#### 2.7.2. Annexins in Plasma Membrane Repair

Annexins A1 and A2 bind to S100A11, which results in actin accumulation to reseal plasma membrane injury in breast cancer cells [185,186]. The buildup of actin is known as a repair cap [187]. Deletion of the Ca^2+^ or annexin binding site of S100A11 disrupts the recruitment of annexin A1/2 and S100A11. Therefore, annexin A1/2 and S100A11 are recruited to the injured site as a complex [185]. Annexins A4, 5, 6, and 7 are also involved in plasma membrane repair [100,188,189].

Annexins A1, 2, 5, and 6 translocate to the wound area in a wave-like manner within 2–30 s after injury and form a repair cap, which is dependent on Ca^2+^ and actin [187,190]. Annexin A5 forms a trimer through self-assembly on PS-exposed membranes [189]. An annexin A5 mutant with a disrupted trimer structure inhibited membrane repair; however, membrane binding was not inhibited [189], suggesting that self-assembly of annexin A5 is required for membrane repair but not membrane binding.

Annexins A4 and A6 regulate membrane curvature [188]. Annexin A4 binds to the edge of the membrane and rolls it up. In contrast, annexin A6 constricts the planar membrane. As observed through live imaging, annexin A6 is localized as a ring-like structure around the wound, whereas annexin A4 rolls up the hole at the edge. Subsequently, annexin A6 constricts to close the membrane [175], which is completed within 15 s [188].

Annexin A7 also plays a role in plasma membrane repair [100]. It binds to ALG2 and recruits ALG2 to the plasma membrane within 100 s. Annexin A7 knockout delays ALG2 recruitment to plasma membrane lesions and inhibits the CHMP4B accumulation required for membrane shedding. CHMP4B is localized to the repair site, and shed vesicles are observed from the plasma membrane within 2–15 min. Annexin A7 recruitment occurs even earlier than ALG2, and annexin A7 cooperatively functions with ALG2 to recruit ESCRT components in a Ca^2+^-dependent manner [100].

#### 2.7.3. Annexins in Endolysosomal Repair

Annexin A2 is recruited to damaged MVBs as well as lysosomes in dendritic cells [191]. Ultrahigh molecular weight polyethylene is released from joint replacements as wear debris and induces inflammation when endocytosed by macrophages and dendritic cells [192]. Wear debris consisting of nanometer-to-micrometer-sized particles is incorporated into MVBs, resulting in the release of lysosomal enzymes, such as cathepsin S and B [191,192]. These observations suggest that internalized wear debris induces damage to MVBs and lysosomes. Annexin A2 is recruited to damaged MVBs and lysosomes, and depletion of annexin A2 results in increased release of cathepsin B and IL-1β [191], suggesting annexin A2 plays a role in membrane repair. Recruitment of annexin A2 to liposomes containing PS and cholesterol is enhanced by elevated Ca^2+^ and acidic pH, suggesting that the release of Ca^2+^ and H^+^ from damaged MVBs and lysosomes may promote annexin A2 recruitment. However, whether a similar Ca^2+^- and pH-dependent mechanism operates during damage to early and recycling endosomes remains unclear (Section 4.3). 

Annexins A1 and A2 function in lysosomal repair following LLOMe treatment in human osteosarcoma U2OS cells [106]. Although multiple annexins are recruited to damaged lysosomes within 10–30 min, only annexins A1 and A2 exhibit lysosomal repair activity. Notably, CHMP4B is recruited to damaged lysosomes even in cells depleted of annexin A1 and A2. Conversely, annexins A1 and A2 are recruited to damaged lysosomes in ALIX and TSG101-depleted cells. These results indicate that the annexin A1- and A2-mediated repair pathway functions independently of the ESCRT machinery. Annexin A1 and A2 recruitment is disrupted by Ca^2+^ chelation, which indicates that their recruitment to damaged lysosomes is also calcium-dependent [106]. Furthermore, Annexins A1 and A2 are preferentially recruited to lysosomes, sustaining larger membrane injuries that permit the release of 10 kDa dextran (4.6 nm in diameter). In contrast, ESCRT components are recruited to lysosomes that retain 10 kDa dextran, suggesting that ESCRT responds to smaller membrane injuries than annexins A1 and A2. 

Annexin A7 is important for lysosomal repair in MCF7 and HeLa cells [193]. Annexin A7 is recruited to damaged lysosomes by LLOMe within 15 min, and depletion of annexin A7 exacerbates Gal-3 recruitment to damaged lysosomes, suggesting that annexin A7 contributes to lysosomal repair. However, although annexin A7 binds to ALG2 [100], CHMP4B recruitment to damaged lysosomes is not affected in annexin A7-depleted cells [193], suggesting annexin A7 functions independently of ESCRT. 

Overall, annexins are recruited to the damaged plasma membrane within 1 min after membrane damage, preceding ESCRT recruitment, whereas they are recruited to damaged lysosomes only after ESCRT has already accumulated. These observations suggest that annexins play distinct roles and are recruited through different mechanisms during repair of the plasma membrane and lysosomes. 

#### 2.7.4. Annexins in Membrane Repair by Fusion of Endosomes/Lysosomes to the Plasma Membrane

The plasma membrane can be resealed by vesicle fusion [194,195,196]. Annexins A1 and A2 are associated with dysferlin [197], and depletion of dysferlin causes loss of repair activity at the damaged plasma membrane in muscle cells [196]. Accumulation of vesicles has been observed at the sites of membrane damage [196,198]. A lack of annexin A2 disrupts injury repair in a cholesterol-dependent manner in muscle cells [199]. It is proposed that annexin A1 and A2 associate with vesicles and dysferlin, and upon plasma membrane damage, Ca^2+^ influx induces vesicle fusion with the damaged plasma membrane for resealing [200]. Resealing by fusion between vesicles/endosomes/lysosomes and the damaged plasma membrane has also been reported in non-muscle cells.

Ca^2+^ ionophore or plasma membrane damage by SLO stimulates MVB fusion to the plasma membrane and releases exosomes in the human colon cancer cell line HCT116 [201]. Exosomes are endosome-derived extracellular vesicles secreted by a wide variety of cells [202]. When MVBs fuse with the plasma membrane, their ILVs are released as exosomes [203,204,205]. The tetraspanin protein CD63 localizes to ILVs of MVBs and is secreted in exosomes [206]. Annexins A2 and A6 are recruited to CD63-positive MVBs in a Ca^2+^-dependent manner [201]. Annexin A6 depletion inhibits MVB fusion and the release of exosomes stimulated by a Ca^2+^ ionophore [201]. Moreover, an antibody against annexin A6 prevents SLO-stimulated exosome release. Similarly, annexin A2 depletion inhibits CD63-positive exosomal release in hepatocellular carcinoma [207]. These results indicate that annexins A2 and A6 mediate the fusion of MVBs to the damaged plasma membrane and release exosomes.

In addition to CD63-positive MVBs, Rab5- or transferrin-positive early endosomes are also reported to fuse with damaged plasma membranes in endothelial cells [208]. Endosomal fusion is dependent on Ca^2+^ and occurs rapidly (1.5 s) after injury. Endosomal fusion occurs around the damaged area and is thought to supply excess membrane to constrict the intact membrane to shed the damaged membrane.

## 3. Endosomal Membrane Damage

In this section, we summarize the methods used to damage endosomes. The rupture of endosomes can be achieved using various methods, and raises endosome-specific issues based on the overviews regarding molecular sensors described in the previous section. 

### 3.1. Chloroquine Damages Late Endosomes and Recruits Gal-9

Chloroquine is a traditional antimalarial medicine [209,210] that damages endosomes. At neutral pH, chloroquine is lipophilic and can enter cells. In acidic compartments, such as the late endosomes, it is protonated and becomes hydrophilic, whereupon it is trapped in the lumen of endosomes. Entrapment and protonation of chloroquine inhibit acidification in endosomes, resulting in water flux from the cytosol to the endosomes and producing osmotic pressure. In addition, chloroquine may insert hydrophobic residues into the endosomal membrane to support endosomal lysis [211,212,213,214].

Chloroquine treatment results in Gal-9 recruitment to the Lamp1- or CD63-positive compartment, suggesting that it damages late endosomes [215]. Gal-9 recruitment to damaged endosomes occurs faster and to a greater extent than Gal-3 or Gal-8 [66,215]. Live imaging revealed that Gal-9 is present in damaged endosomes within 3 to 15 s, and the maximum recruitment of Gal-9 to damaged endosomes occurs approximately 3 h after chloroquine treatment [215].

Rab5 is a Rab GTPase that localizes to and mediates the fusion of early endosomes [216,217,218]. Rab5 is recruited to Gal-9-positive endosomes, rather than Gal-9 being recruited to Rab5-positive early endosomes [215]. Therefore, Rab5 may play a role in damaged late endosomes after Gal-9 recruitment.

### 3.2. Chloroquine Damages Early/Recycling Endosomes, and Rabs/Rab Binding Proteins Promote Autophagy of Endosomes

Early and recycling endosomes may be damaged by chloroquine [219]. Rabaptin-5 binds to Rab5 and Rab4 and localizes to early and recycling endosomes [220,221,222,223]. Chloroquine treatment induces increased recruitment of Rabaptin-5 to transferrin receptor (TfR)-positive early/recycling endosomes within 15 min [219], although TfR may also be localized to MVBs [205]. Chloroquine promotes the recruitment of Gal-3 and Gal-8 to Rabaptin-5-positive compartments within 15 min and the recruitment of autophagy-related proteins, ubiquitin, p62, FIP200, and ATG16L, within 30 min [219]. LC3 was observed 150 min after chloroquine treatment. Rabaptin-5 binds to the autophagy-initiating factors FIP200 and ATG16L [219]. Rabaptin-5 depletion inhibits LC3 recruitment to early or recycling endosomes [219], suggesting that it promotes the autophagy of damaged endosomes. In addition, Rabaptin-5 depletion increases the survival of *Salmonella-infected* HeLa and HEK293A cells, suggesting that it contributes to the restriction of *Salmonella* proliferation. ALIX and CHMP4B are not detectable 30 min after chloroquine treatment [219]. Chloroquine treatment may induce severe effects on endosomes, and the repair process by ESCRT may be bypassed before detection (Section 3.3 and Section 3.6).

Recent studies have indicated that multiple Rab GTPases interact with autophagy receptors, predominantly NDP52, but also p62 [224]. Rabs and their binding partners may be involved in activating autophagy pathways rapidly upon endosomal damage.

### 3.3. Hypertonic/Hypotonic Shock in Endosomes

Hypertonic shock applied with 0.5 M sucrose and 10% polyethylene glycol (PEG) 1000 to the L929 mouse fibroblast-like cell line for 10 min, followed by hypotonic shock with 60% medium in water, disrupts pinocytic vesicles by osmotic lysis [225]. Internalized horseradish peroxidase (HRP) is maintained in pinocytic vesicles or early endosomes during hypertonic shock, and subsequent hypotonic shock releases HRP into the cytosol [225]. This procedure recruits Gal-3, -8, and -9 as cellular puncta [57]. Therefore, hypertonic/hypotonic shock may induce endosomal membrane damage.

Lysenin is recruited to damaged endosomes within 20 min following osmotic shock [58]. The endogenous receptor of exposed sphingomyelin, TECPR1, is also recruited [121], suggesting that sphingomyelin exposure occurs during endosomal damage. While it is tempting to speculate that nSMase and ceramide are involved in the endosomal repair process, as in the plasma membrane and lysosomes, direct evidence for their role in endosomal membrane repair remains lacking. Rather, TECPR1 is more implicated in inducing autophagy [121,127]. Whether these sphingolipids actively participate in endosomal repair requires future work.

Hypertonic shock recruits the ESCRT machinery, ALIX, CHMP4B, and CHMP1B to early/late endosomes and lysosomes [226]; however, the CHMP4B signal decreases following hypotonic shock. Endosomal integrity is intact under hypertonic solution, and replacing a hypertonic solution with a hypotonic or isotonic solution likely restores endosomes to normal conditions. Therefore, ESCRT recruitment is not triggered by membrane damage under hypertonic conditions. Hypertonic shock promotes water efflux from the cells to the extracellular space, resulting in lower membrane tension [227,228]. Low membrane tension induced by EGF internalization also promotes ESCRT recruitment, suggesting that ESCRT assembly on endosomes and lysosomes is triggered by excess membrane to promote ILV formation, rather than membrane damage.

### 3.4. Cationic Nanoparticles Disrupt Phagosomes and Endosomes

Cationic lipids are widely used for intracellular delivery of nucleic acids and are known to damage endosomal membranes. They interact with negatively charged phospholipids in biological membranes [229,230,231]. After internalization of cationic lipid-coated polystyrene beads for 1 h, LC3 is detectable, suggesting that phagosomes are damaged, leading to autophagy induction [46,232]. Cationic magnetic nanoparticles, after 1 h of internalization, colocalize with LC3 in early/recycling endosomes positive for Vps26, indicating that early/recycling endosomes are damaged. Calcium phosphate precipitates (CPP) complexed with DNA internalized for 4 h recruit Gal-3, which colocalizes with Rab7 and Lamp1, suggesting that damaged organelles are late endosomes or lysosomes [233]. LC3 is recruited to the lysosomes after CPP internalization, and the depletion of Gal-3 results in a reduction in LC3. Therefore, CPP targets late endosomes/lysosomes for lysophagy through Gal-3. Cationic polymers, such as polyethyleneimine (PEI), permeabilize the plasma membrane and cause toxicity [234,235]. Collectively, these findings suggest that cationic cargoes can cause membrane damage that exceeds the repair capacity of phagosomes, endosomes, or lysosomes, thereby triggering LC3 recruitment and selective autophagy.

### 3.5. Ionizable Lipid Nanoparticles (LNPs) Disrupt Endosomes

Ionizable lipids are neutral at physiological pH but become cationic under endosomal pH by protonation [236,237]. Ionizable lipids interact with anionic phospholipids only in the endosomal lumen and destabilize the endosomal membrane [238]. Lipid nanoparticles (LNPs) consist of ionizable lipids, cholesterol, and helper lipids and are typically 50–100 nm in diameter. LNPs have been clinically approved for RNA delivery, including mRNA vaccines against COVID-19 [236].

Lipoplexes are complexes of ionizable lipids and RNAs. RNA release has been visualized by a temporal increase in fluorescent RNA from fluorescence-quenching conditions in endosomes [239]. Lipoplexes are delivered to EEA1-positive early endosomes, and RNAs are released just after EEA1 disappearance from endosomes [239]. A dominant negative Rab7 mutant or inhibitors of lysosomal acidification inhibit the knockdown efficiency of siRNAs; endosomal acidification is required for siRNA release [239]. LC3 is recruited approximately 8 min after RNA release, suggesting that endosomal damage triggers autophagy. Endosomal damage by lipoplexes can also be detected by Gal-8, Gal-9, and, less efficiently, by Gal-3 [239]. Depletion of Gal-8 or Gal-9 prevents LC3 recruitment, suggesting that Gal-8 and Gal-9 trigger the autophagy pathway. However, depletion of Gal-8 or Gal-9 does not inhibit siRNA knockdown efficiency; therefore, the autophagy pathway does not inhibit siRNA knockdown [239]. Ca^2+^ release is also detectable near endosomes by lipoplex. Ca^2+^ increase is coincident with RNA release, though not all endosomes containing the lipoplex cause Ca^2+^ release [239].

Endosomal damage and RNA release by LNPs have been studied [215,239,240,241], although the efficiency of endosomal escape of RNAs by LNPs is very low (1–2%) [25]. Endosomal damage by LNPs can be detected by Gal-3, -8, and -9 [66,239] and TECPR1 [127]. After internalization, LNPs recruit Gal-8 and Gal-9 [66]. RNA release occurs after EEA1 disappearance but before Rab7 recruitment, suggesting that LNPs damage maturing endosomes [239]. However, endosomal escape of mRNA from recycling endosomal tubules has also been observed [240], though the majority of cargoes reside in late endosomes or lysosomes. Interestingly, *Shigella* also uses recycling endosomal Rabs, such as Rab11, Rab8, and Rab35, for its escape from BCVs [242,243,244]. *Shigella* promotes macropinocytosis independently of phagosomes, and the fusion of macropinosomes with BCVs appears to play an important role in *Shigella* escape from BCVs [243,244,245]. It remains unclear which properties of endosomes facilitate endosomal escape, such as tubular morphology, membrane composition, or luminal acidification, or whether multiple endosomal populations contribute to membrane escape.

### 3.6. Specific Endosomal Damage Using Physical Energy

We reported that internalization of 20 nm of magnetic nanoparticles by MCF7 cells, followed by application of a direct current magnetic field at 100 mT for 5 min, induces endosomal damage [246]. Under these conditions, CHMP4B and Gal-3 recruitment were observed in EEA1-positive early endosomes, suggesting that they are injured. As LC3 recruitment was not detected under this condition [246], Gal-3 and the ESCRT machinery may repair endosomes; therefore, LC3 recruitment is not required.

We estimated the force generated per nanoparticle as ~fN level based on the speed of 1 μm magnetic nanoparticles under these conditions. Even if 1000 magnetic nanoparticles accumulate within an endosome, the total generated force would remain below 100 pN, which is lower than the ~500 pN reported for plasma membrane rupture by AFM [247]. However, as Gal-3 is recruited, MNPs may damage endosomes locally.

When magnetic nanoparticles accumulate in the whole luminal area of endosomes, the endosomal shape may be deformed by a magnetic field greater than 20 mT for 15 min [248]. Membrane disruption was not described in this case; however, the partial accumulation of magnetic nanoparticles in endosomes may damage or permeabilize the membrane locally.

Physical approaches have been developed to enable fine-tuning of local damage to intracellular organelles. Internalization of the photosensitizer, disulfonated aluminum phthalocyanine (AlPcS_2a_), with illumination of 635 nm light, damaged lysosomes [249]. Internalization of barium titanate with the application of ultrasound damaged lysosomes and induced the differentiation of M1 macrophages [250].

## 4. Models of the Initial Events of Membrane Damage

The key molecules involved in membrane damage sensing are listed in Table 1 together with their approximate recruitment times. Recruitment kinetics vary depending on the cell type and experimental conditions. In addition, advances in imaging technologies may enable the detection of membrane damage at much earlier time points. Therefore, readers should not interpret these data as representing a single unified sequence of molecular events. Nevertheless, side-by-side comparison of the currently available recruitment kinetics provides a useful framework for understanding the order of events following membrane damage, including damage sensing, membrane repair, and clearance of damaged organelles, by highlighting which molecules are recruited during the early and late stages.

In general, plasma membrane damage causes the recruitment of sensor molecules considerably faster than lysosomal membrane damage, although not all molecules are involved in both pathways. This is consistent with the concept that plasma membrane damage is more toxic to host cells and requires more rapid repair compared with lysosomal damage; however, the threshold of membrane damage that cannot be repaired and progresses to cell death or clearance of organelles remains unclear. 

To compare the processes among the plasma membrane, lysosomes, and endosomes, we summarize the initial events in the next section.

### 4.1. Initial Phase of Plasma Membrane Damage

**Figure 1:**
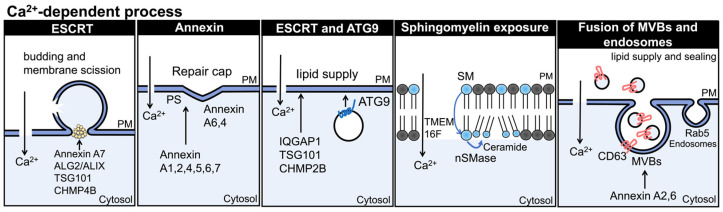
**Schematic summary of the initial phase of plasma membrane damage.** Plasma membrane damage provokes Ca^2+^ influx, leading to ESCRT, annexins, and ATG9 vesicle recruitment. Ca^2+^ influx also induces sphingomyelin exposure to the cytosol and endosomal fusion. For more detailed molecular models, readers are referred to the relevant sections of this review and the cited original articles.

The events during the initial phase of plasma membrane damage are summarized in Figure 1. To date, all the processes described in this initial phase appear to be Ca^2+^-dependent, although Ca^2+^-independent mechanisms may be identified in future studies. These responses occur rapidly, typically within 1 s to 1 min (Table 1). Molecules recruited to plasma membrane lesions in a Ca^2+^-dependent manner usually participate in membrane repair. We also included fusion between the plasma membrane and endosomes, as fusion with early endosomes has been reported to occur as early as 1.5 s after injury (Section 2.7.4). However, it remains unclear whether endosome–plasma membrane fusion is a universal mechanism or is restricted to specific cell types, and whether specialized endosomal or lysosomal populations are dedicated to plasma membrane resealing.

### 4.2. Initial Phase of Lysosomal Damage

**Figure 2:**
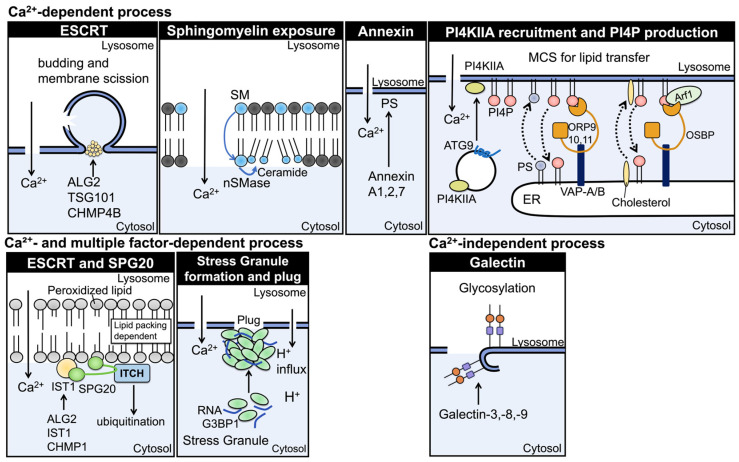
**The schematic summary of the initial phase of lysosomal damage.** Lysosomal damage also causes Ca^2+^ flux. Recruitment of ESCRT, annexins, PI4KIIA, and sphingomyelin exposure is dependent on Ca^2+^. Ca^2+^ with lipid peroxidation recruits SPG20, and Ca^2+^ and H^+^ influx may induce stress granule formation. Galectin recruitment is Ca^2+^-independent. For more detailed molecular models, readers are referred to the relevant sections of this review and the cited original articles.

The events during the initial phase of lysosomal damage are summarized in Figure 2. Lysosomal damage also induces Ca^2+^-dependent processes similar to those observed during plasma membrane repair. However, both Ca^2+^-dependent and -independent pathways appear to operate during lysosomal damage. SPG20 may be recruited to damaged lysosomes through both IST1 dependently of Ca^2+^ and peroxidized lipids independently of Ca^2+^ (Section 2.2.5). Stress granule formation may also be regulated by Ca^2+^ and H^+^ (Section 2.4). In contrast, galectins are recruited independently of Ca^2+^, as they recognize intralumenal glycans exposed upon membrane damage (Section 2.1.3). Overall, Ca^2+^-dependent pathways appear to primarily respond to small membrane lesions and promote membrane repair, whereas Ca^2+^-independent pathways are more strongly associated with larger lesions and autophagy, although Gal-3 may also contribute to membrane repair (Section 2.1.3). Ca^2+^ release from small membrane lesions may represent one of the earliest events following lysosomal damage. Whether the transition from membrane repair to autophagy is governed by lesion size, the persistence of membrane damage, or both remains unclear.

### 4.3. Endosomal Damage

**Figure 3:**
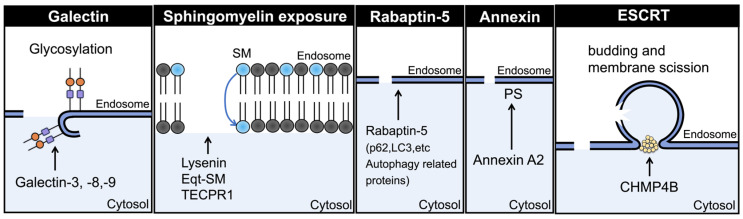
**Schematic summary of the initial phase of endosomal damage.** In damaged endosomes, galectins, annexins, and Rabaptin-5 are recruited. Sphingomyelin exposure also occurs. For more detailed molecular models, readers are referred to the relevant sections of this review and the cited original articles.

The endosomal damage response is summarized in Figure 3. The role of Ca^2+^ in downstream responses to endosomal damage remains unclear. Upon endosomal damage, sphingomyelin exposure, annexin recruitment, and ESCRT recruitment have been observed (Section 2.7.3, Section 3.5 and Section 3.6). In addition, a Ca^2+^ increase around lipoplexes has been observed (Section 3.5). These findings suggest that molecular events typically associated with Ca^2+^ signaling during plasma membrane and lysosomal repair may also contribute to the response to endosomal membrane damage. However, the Ca^2+^ concentration in endosomes is lower than that in lysosomes or the extracellular space [251]. Annexin A2 and ESCRT can be recruited to early endosomes independent of Ca^2+^ (Section 2.7.1 and Section 3.3). Rabaptin-5 is also localized in early and recycling endosomes, where it normally functions in membrane fusion rather than specifically in the response to endosomal damage (Section 3.2). Unlike plasma membrane and lysosomal repair, endosomal membrane repair may preferentially utilize membrane trafficking machinery that normally functions in endosomal trafficking, rather than relying on Ca^2+^-dependent recruitment of repair machinery. Endosomal damage has been efficiently detected by Gal-3, -8 and -9 (Section 3.1, Section 3.5 and Section 3.6).

It remains unclear whether cargo release from endosomes always requires membrane damage sufficient to induce LC3 recruitment. When endosomal damage exceeds the membrane repair capacity, autophagy is activated. However, whether cargo release can occur following milder membrane damage without autophagy induction remains unknown. The threshold may also depend on cargo size.

Which endosomal populations are most permissive for cargo escape remains unclear. Both acidic and recycling endosomes have been implicated in endosomal escape (Section 3.5). However, the membrane properties that facilitate escape remain to be elucidated.

## 5. Conclusions

Studies on bacterial infection and organelle membrane damage have significantly advanced our understanding of how cells detect sterile membrane damage. We summarized the molecules that can detect membrane damage during the initial phase. These molecules mostly act as damage sensors and are involved in subsequent membrane repair. When damage is prolonged without repair, downstream events, such as the autophagic process of damaged organelles, may be activated. Membrane fusion between the plasma membrane and endosomes or lysosomes for membrane repair also plays an important role. These processes should be further elucidated in the future. Studies on membrane damage provide insights into the methods of gene transfection and foster the development of a novel physical energy-based approach to precisely control membrane damage. Continued discoveries in this field are expected to expand our knowledge regarding cellular events as well as the development of novel technologies that contribute to a wide range of biomedical research.

## Figures and Tables

**Table 1:** **Molecules recruited to damaged organelles during the initial phase.** The “Recruited organelle” column indicates the organelles to which each molecule localizes upon membrane damage. The “Timing” column indicates the reported timing of recruitment after membrane damage. The reported timings are based on representative studies and may vary depending on the experimental conditions. Therefore, the reported timings should not be interpreted as indicating a single universal sequence of molecular recruitment. Nevertheless, they highlight the approximate differences in the temporal dynamics of membrane damage responses among different molecules and organelles.

Molecule	Recruited Organelle	Timing	References
Galectin-3	Lysosomes, endosomes	5–20 min	[34,35,46,47,49,246]
Galectin-8	Lysosomes, endosomes	10–15 min	[57,58]
Galectin-9	Lysosomes, endosomes	15 s–3 h	[61,66,215,241]
LC3	Lysosomes, endosomes	30 min	[34,35,46,47,57]
Ubiquitin	Plasma membrane, lysosomes, endosomes	6 min 60–120 min	[55,90,101]
UBE2QL1	Lysosomes	30 min	[52]
ALIX	Plasma membrane, lysosomes	30 s 10 min	[37,55,90,91,101,226]
ALG2	Plasma membrane	30 s	[91,92,100]
CHMP4B	Plasma membrane, lysosomes, endosomes	10–60 s 10 min	[37,55,90,91,101,226]
IST1	Lysosomes	60 s	[51,107]
SPG20	Lysosomes	5 min	[51]
ITCH	Lysosomes	15 min	[51]
^a^ Lysenin	Lysosomes, endosomes	10–15 min	[58,116]
^a^ EqtSM	Plasma membrane, lysosomes	5 s 5 min	[49,119]
TECPR1	Endosomes, lysosomes	10–20 min	[121,122]
G3BP1/2	Stress granules plug damaged lysosomes	3–20 min	[128,129,130]
Rabaptin-5	Endosomes	30 min	[219]
PI4KIIA	Lysosomes	10 min	[137]
^a^ SidM	Lysosomes	5–10 min	[142,168]
ORP9-11	Lysosomes	10 min	[137]
OSBP-PH	Lysosomes	30 min	[137,142]
ATG2A	Lysosomes	10 min	[137]
ATG9A	Lysosomes, plasma membrane	15–45 min 1–10 min	[168,169]
Annexins A1, 2, 4, 5, 6, 7 Annexins A1, 2, 7	Plasma membrane, MVBs, lysosomes,	2–30 s 10–30 min	[100,106,187,188,189,190,191,193,201]

^a^ Exogenous probes for membrane damage detection, rather than endogenous cellular proteins (see text).

## Data Availability

No new data were created or analyzed in this study. Data sharing is not applicable to this article.
